# SHQ1-associated neurodevelopmental disorder: Report of the first homozygous variant in unrelated patients and review of the literature

**DOI:** 10.1038/s41439-023-00234-z

**Published:** 2023-02-22

**Authors:** Aljouhra AlHargan, Mohammed A. AlMuhaizea, Rawan Almass, Ali H. Alwadei, Maha Daghestani, Stefan T. Arold, Namik Kaya

**Affiliations:** 1 Translational Genomics Department, MBC: 26, Center for Genomic Medicine, King Faisal Specialist Hospital and Research Centre (KFSHRC), Riyadh, 11211 Saudi Arabia; 2 Department of Zoology, P.O. Box. 145111, College of Sciences, King Saud University, Riyadh, 11362 Saudi Arabia; 3 Neuroscience Centre, MBC: 76, King Faisal Specialist Hospital and Research Centre, Riyadh, 11211 Saudi Arabia; 4 College of Medicine, AlFaisal University, Riyadh, Saudi Arabia; 5 Department of Medical Genomics, MBC: 75, Center for Genomic Medicine, King Faisal Specialist Hospital and Research Centre, Riyadh, 11211 Saudi Arabia; 6 Pediatric Neurology Department, National Neuroscience Institute, King Fahad Medical City, Riyadh, Saudi Arabia; 7 Bioscience Program, Biological and Environmental Science and Engineering Division, King Abdullah University of Science and Technology (KAUST), Thuwal, 23955-6900 Kingdom of Saudi Arabia; 8 Computational Biology Research Center, King Abdullah University of Science and Technology, Thuwal, Saudi Arabia; 9 Centre de Biologie Structurale (CBS), INSERM, CNRS, Université de Montpellier, F-34090 Montpellier, France

**Keywords:** Neurodegeneration, Neurodegeneration

## Abstract

Compound heterozygous mutations in *SHQ1* have been associated with a rare and severe neurological disorder characterized by global developmental delay (GDD), cerebellar degeneration coupled with seizures, and early-onset dystonia. Currently, only five affected individuals have been documented in the literature. Here, we report three children from two unrelated families harboring a homozygous variant in the gene but with a milder phenotype than previously described. The patients had GDD and seizures. Magnetic resonance imaging analyses revealed diffuse white matter hypomyelination. Sanger sequencing confirmed the whole-exome sequencing results and revealed full segregation of the missense variant (*SHQ1*:c.833 T > C; p.I278T) in both families. We performed a comprehensive in silico analysis using different prediction classifiers and structural modeling of the variant. Our findings demonstrate that this novel homozygous variant in *SHQ1* is likely to be pathogenic and leads to the clinical features observed in our patients.

## Introduction

Shq1 has been identified in yeast during the biogenesis of H/ACA ribonucleoproteins (RNPs)^1^ and has been found to interact with one of the catalytic subunits of the RNPs, dyskerin/NAP57. This molecule is a critical assembly factor for H/ACA ribonucleoproteins (RNPs)^2^. Therefore, it is involved in various important functions, such as telomerase maintenance, ribosomal modifications, protein translation, and pre-mRNA splicing^2,3^. In humans, *SHQ1* has similar functions and stabilizes the accumulation of H/ACA RNPs by binding with *NAP57* at an early stage, which promotes the biogenesis of H/ACA RNPs^4^. Accordingly, loss of *SHQ1* will lead to degradation of the RNP assembly^5^. Mutations in genes encoding components of the H/ACA RNP complex, such as DKC1, can lead to significant effects on neurological development^6^. Recently, two studies reported patients with pathogenic variants of *SHQ1* with neurological disorders; Bizarro and Meier^7^ reported singletons with intrauterine growth retardation coupled with a severe-onset neurological disease inclusive of cerebellar degeneration, whereas Sleiman et al.^6^ studied two separate families harboring four patients (two individuals in each family) with early-onset dystonia, hypotonia, seizure disorder, and global developmental delay (GDD)^6^. Interestingly, all reported variants in both studies were compound heterozygous^6,7^. Here, we report three individuals from two unrelated families with a novel homozygous variant in *SHQ1* and show that the variant is likely to be pathogenic. Informed consent was obtained from all subjects enrolled in the study. The patients were recruited from the pediatric neuroscience clinic. Peripheral blood samples were collected, and genomic DNA was isolated from whole blood using the Gentra® Puregene® DNA Purification Kit (Gentra Systems, Inc. Minneapolis, MN, US). Axiom-based genotyping was conducted using Affymetrix axiom chips followed by autozygome analysis. In addition, whole-exome sequencing (WES) variant calls were performed by an Illumina 2500 platform using libraries that were prepared by a SureSelect kit (Illumina Inc., San Diego, CA, US). WES data were filtered, and low-frequency pathogenic variants were targeted according to previously published protocols. Confirmatory sequencing and segregation were performed by Sanger sequencing using an ABI PRISM 3100 Genetic Analyzer (Applied Biosystems, Foster City, CA, USA). The structure of the C-terminal domain of SHQ1 was modeled using Swiss-Model based on the 30% identical yeast homolog (PDB ids 3zv0, 3zuz; QMEAN 0.41). The homology model was corroborated by the AlphaFold2 model for this region (RMSD 1.12 Å; pLDDT > 90 for the core region).

### Family 1, Patient IV:1

**Fig. 1:**
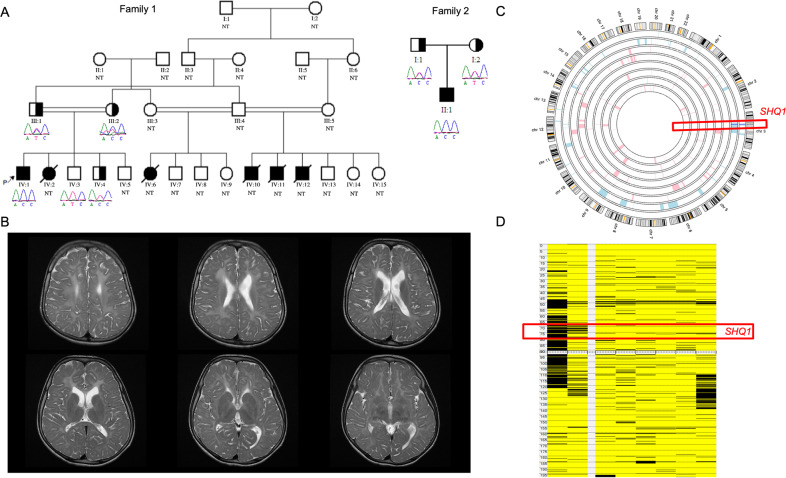
Genetic analyses of the families. **A** Pedigrees present two unrelated families with carrier parents and three affected individuals in total, in addition to a healthy carrier brother and two normal brothers in Family 1 and four affected deceased parental cousins. Brief chromatograms of the variant are shown below each symbol representing the family members. NT = not tested. Half-filled symbols indicate the carrier status of an individual, whereas completely filled symbols (in black color) refer to the affected individuals. The full chromatograms are presented in Supplementary Fig. 1. The black arrow points to the index case for the study. **B** Brain MRI of a patient with the *SHQ1* variant (Family 1, Patient IV:1) showing severely delayed myelination generally in mildly prominent CSF spaces. **C** Autozygosity analysis using Agile multi-ideogram revealed a single shared run of homozygosity between the affected individuals on chromosome 3. **D** The same analysis using AutoSNPa confirmed the shared runs of homozygosity on chromosome 3.

Individual 1 (Fig. 1A, Family 1, Patient IV:1) is a 15-year-old male, who was born after a full-term pregnancy by normal spontaneous delivery, from a first-degree consanguineous Saudi family. He was admitted after birth to the NICU due to jaundice and low birth weight. Patient IV:1 was first diagnosed at 5 years of age with global developmental delay (GDD), ataxia and seizure disorder. Brain MRI showed severely delayed myelination (Fig. 1B). His developmental delay involved motor, cognition, and speech. By age 15, he was able to walk with support and had very limited speech output. His seizures were controlled by one medication. His examination was notable for mild spasticity in the lower limbs and brisk deep tendon reflexes.

### Family 1, Patient IV:2

Individual 2 (Fig. 1A, Family 1, Patient IV:2) is the younger sister of Patient IV:1. She was born after a full-term pregnancy by normal spontaneous delivery and was admitted to the NICU due to jaundice. She had a similar diagnosis as her brother with GDD and seizure disorder. She also had generalized hypotonia and was wheelchair-bound. Brain MRI showed diffuse white matter hypomyelination. Patient IV:2 died at the age of 9 years due to cardiac arrest. The patient’s father reported that Patient IV:2 deteriorated in her last days; she was in a vegetative state and had severe malnutrition.

Individuals 1 and 2 (Fig. 1A, Family 1) had a positive family history of similar illness with four parental cousins diagnosed with GDD, all of whom had passed away.

### Family 2, Patient II:1

Individual 3 (Fig. 1A, Family 2, Patient II:1) is a 7-year-old male of a Saudi family who was delivered by C-section late preterm with low birth weight and was admitted to the hospital after birth. He was diagnosed with GDD, seizure disorder and ataxia in addition to frontotemporal atrophy. At approximately 4 years old, the patient developed neutropenia and thrombocytopenia.

**Fig. 2:**
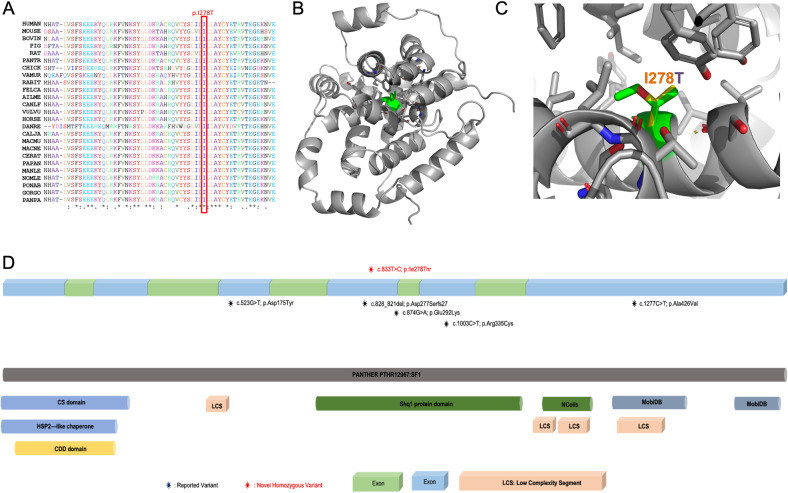
Structural analysis of the human SHQ1 C-terminal domain. **A** Amino acid sequences of SHQ1 belonging to various species were downloaded from Ensembl^9^ and aligned using Clustal Omega from EBI. **B** The modeled structure of SHQ1 residues 143 to 421 is shown as a gray ribbon. I278 is shown in green. **C** Neighboring side chains are shown as stick models with carbon in gray, oxygen in red and nitrogen in blue. I278 is shown in green, and the corresponding threonine is shown with carbons in yellow and oxygen in red. **D** Previously reported variants and the novel homozygous p.Ile278Thr variant are presented on a schematic drawing of the gene and protein. Alternating boxes (light green and blue color) represent exons (upper part of the panel). The lower part of the panel includes the protein domains of SHQ1 according to Ensembl^9^.

WES filtering revealed that the patients in both family 1 and family 2 had the same novel homozygous missense variant (c.833 T > C; p.I278T) in *SHQ1* (Fig. 1A). The homozygosity scan using Affymetrix’s axiom SNP arrays showed that *SHQ1* is shared in the same runs of the homozygosity block on chromosome 3 (Fig. 1C, D). The variant c.833 T > C has a low frequency (1000 G = 0/GnomAD = 0) with in silico pathogenicity classifiers, indicating that the variant is likely highly pathogenic (SIFT = 0.001, damaging; PolyPhen = 1, probably damaging; VEST = 0.933 and 0.925, deleterious; PP2HVAR (PolyPhen 2) = 0.99, probably damaging; PP2HDIV = 1, probably damaging; MutationTaster = 1,1, disease causing; MutationAssessor, 3.025 medium, predicted functional). The variant is located in a highly conserved region of the C-terminal domain of SHQ1 (Fig. 2A). The three-dimensional structure of this domain can be inferred based on the known structures of the yeast homolog. I278 is buried in the hydrophobic core of this domain (Fig. 2B). Its substitution by a smaller and polar threonine will introduce a polar moiety and a spatial gap in the protein core (Fig. 2C). Hence, the p.I278T mutation will destabilize this domain and affect its function, specifically, acting as an assembly chaperone that protects Cbf5 protein complexes from nonspecific RNA binding and aggregation before assembly of H/ACA RNA^8^.

The first compound heterozygous variants (*SHQ1:* c.1003 C > T; p.Arg335Cys/c.1277 C > T; p.Ala426Val) were reported in Bizarro and Meier’s study in a male patient who was delivered by an urgent cesarean section owing to intrauterine growth retardation and sparse fetal movement. The patient was born at gestational week 29 with a body weight of 1000 g^7^. Since birth, he had GDD in all milestones in addition to enormous feeding difficulties. At early ages, he developed cerebellar hypoplasia with clearly recognizable atrophy and ventricular dilatation in addition to epileptic seizures. On the last examination, he was in a vegetative condition with cortical atrophy and spastic quadriplegia^7^.

The second report (Sleiman et al.)^6^ examined four patients in two different families, apparently unrelated^6^. Although the compound heterozygous variants (SHQ1: c.874 G > A; p.Glu292Lys/c.828_831del; p.Asp277SerfsTer27, Family 1; c.523 G > T; p.Asp175Tyr/c.828_831del; p.Asp277SerfsTer27, Family 2) were different in each family, one of the alleles, a deletion (c.828_831del), was shared^6^. Interestingly, all the variants were located within the SSD domain of SHQ1. Patients in both families were born at term, and their blood work showed normal biochemistry. In Family 1, both affected individuals had a similar onset that started at birth and was characterized by developmental delay and dystonia, whereas the affected individuals of Family 2 were only described as having a movement disorder. Although the first MRI scans of the patients in both families were normal, a second MRI was performed on Patient IV:1 of Family 1 at the age of 4 years and showed subtle atrophy of the cerebellum accompanied by widening of the cerebellar folia. All patients in this report were treated with levetiracetam in addition to levodopa replacement therapy, but therapy was discontinued in Patient II:1 of Family 2 because it caused dyskinesia in the patient^6^.

Similarly, our patients were born at term except for Patient II:1 of Family 2 (born late preterm) and had normal biochemistry and low birth weight. However, unlike previously reported cases^6,7^, our patients showed a homozygous allelic variant in *SHQ1*. All affected individuals in our study as well as the previously reported cases^6,7^ were diagnosed with GDD, and most also had a seizure disorder. One of our patients (Patient IV:2) and one patient of Sleiman et al.^6^ additionally had hypotonia. Table 1 summarizes the clinical features of our patients and the previously reported cases of *SHQ1* mutations.

**Table 1:** List of *SHQ1* reported cases with comprehensive delineation of clinical details.

		This study			Sleiman, 2021				Bizarro, 2017
Patients’ Info.	Family #	1	1	2	1	1	2	2	1
	Individual	IV:1	IV:2	II:1	1	2	3	4	1
	Gender	Male	Female	Male	Female	Female	Male	Male	Male
	Age (years)	15	Died at 9	7	Died at 10	11	10	8	ND
	Origin	Saudi	Saudi	Saudi	European	European	Caucasian	Caucasian	ND
	Consanguineous	Yes	Yes	ND	No	No	No	No	ND
	Family history	Yes	Yes	ND	No	No	No	No	ND
	Current state	Alive	Deceased	Alive	Deceased	Alive	Alive	Alive	Alive
WES	Mutation type	HMZ	NT	HMZ	Compound HTZ	Compound HTZ	Compound HTZ	Compound HTZ	Compound HTZ
	c.DNA	c.833 T > C	NT	c.833 T > C	c.874 G > A / c.828_831del	c.874 G > A / c.828_831del	c.523 G > T / c.828_831del	c.523 G > T / c.828_831del	c.1003 C > T / c.1277 C > T
	Protein	p.I278T	NT	p.I278T	p.E292K / p.D277SfsX27	p.E292K / p.D277SfsX28	p.D175Y / p.D277SfsX27	p.D175Y / p.D277SfsX28	p.R335C / p.A426V
Phenotypes	Birth	Born at term	Born at term	Late pre-term	Born at term	Born at term	Born at term	Born at term	Very pre-term
	Birth weight	Low birth weight	Normal	Low birth weight	73rd percentile	74th percentile	75th percentile	90th percentile	Low birth weight
	Age at diagnosis	5 years	2 years	4 years	<6 months	<6 months	<6 months	<6 months	Birth
	Mobility	Supported walking	Vegetative state	ND	Wheel-chair	Wheel-chair	ND	ND	Vegetative state
	GDD	Yes	Yes	Yes	Yes	Yes	No	No	Yes
	Hypotonia	No	Yes	ND	Yes	Yes	No	No	ND
	Dystonic/ataxic gait	Yes	ND	ND	Yes	Yes	Yes	Yes	ND
	Seizure	Yes	Yes	Yes	Yes	Yes	No	No	Yes
	Intellectual delay	Yes	Yes	Yes	ND	ND	No	No	ND
	Speech delay	Yes	Yes	ND	ND	ND	ND	ND	ND
Other findings	Medication	Levetiracetam	Levetiracetam	ND	Levetiracetam	Levetiracetam / Levodopa / Carbidopa	Levodopa / Trihexyphenidyl	Levodopa	ND
	Biochemistry	Normal	Normal	ND	Normal	Normal	Normal	ND	Normal
	MRI	Delayed myelination	Diffused white matter and hypomyelination	Frontotemporal atrophy	Subtle atrophy of the cerebellum with widening of the cerebellar folia	Normal	Normal	Normal	Cerebellar hypoplasia, ventricular dilatation and marked atrophy.

*ND* no data, *HMZ* homozygous, *NT* not tested, *HTZ* heterozygous.

It is noteworthy to mention that our patients presented with a milder phenotype than the patients reported by Sleiman et al.^6^ and Bizarro and Meier^7^. Our patients achieved the ability to walk with ataxic gait, were able to produce single but few words and used their hands to play with smartphones. Their understanding was that of a 5- to 6-year-old when they were 14 years old. In addition, they had milder spasticity more noticeable in the lower limbs with brisk reflexes, and the proband with the longest follow-up remained ambulant until the last visit at age 16. The spasticity was very mild and never impaired ambulation. The patients also had limb ataxia. Ataxia was the greatest contributor to recurrent falls. These findings may be explained by the cerebellar atrophy noted during autopsy studies and on MRI showing cerebellar and supratentorial atrophy. Apart from that, there was no clear degenerative course or decline in the proband’s abilities apart from dysphagia. The seizures were easily controlled with a single medication. The movement disorder was characterized by hyperkinetic choreiform movements. Moreover, our patients did not display florid dystonia or ballismus, as reported by Sleiman et al^6^. Due to the atypical phenotype of our patients, comprehensive evaluation was otherwise unremarkable, including plasma amino acids, renal profile, uric acid, lactate, ammonia, transferrin isoelectric focusing, CK and liver function tests, tandem MS, very long-chain fatty acids, urine organic acids, serum copper and ceruloplasmin. Moreover, the leukodystrophy next-generation sequencing panel and SNP array for cytogenetic analysis were normal. In contrast, the patients reported by Sleiman et al.^6^ and Bizarro and Meier^7^ were averbal with severe dystonia and autonomic instability and were wheelchair bound with normal MRI imaging. Hence, our cases represent a milder phenotype. We suspect that such clinical differences may be due to phenotypic variability.

In conclusion, our study reports the first *SHQ1* homoallelic variant in three patients from two separate unrelated families who presented with global developmental delay, ataxia, and seizure disorder.

## HGV Database

The relevant data from this Data Report are hosted at the Human Genome Variation Database at 10.6084/m9.figshare.hgv.3279.

### Supplementary information

Supplementary figure 1.

Supplementary figure 1 legend

## Data Availability

The data that support the findings of this study are available from the corresponding author upon reasonable request.
