# Supplementary figures and images for: SHQ1-associated neurodevelopmental disorder: Report of the first homozygous variant in unrelated patients and review of the literature
Source: Hum Genome Var. 2023 Feb 22;10:7. doi: 10.1038/s41439-023-00234-z (PMC9944922; doi:10.1038/s41439-023-00234-z)

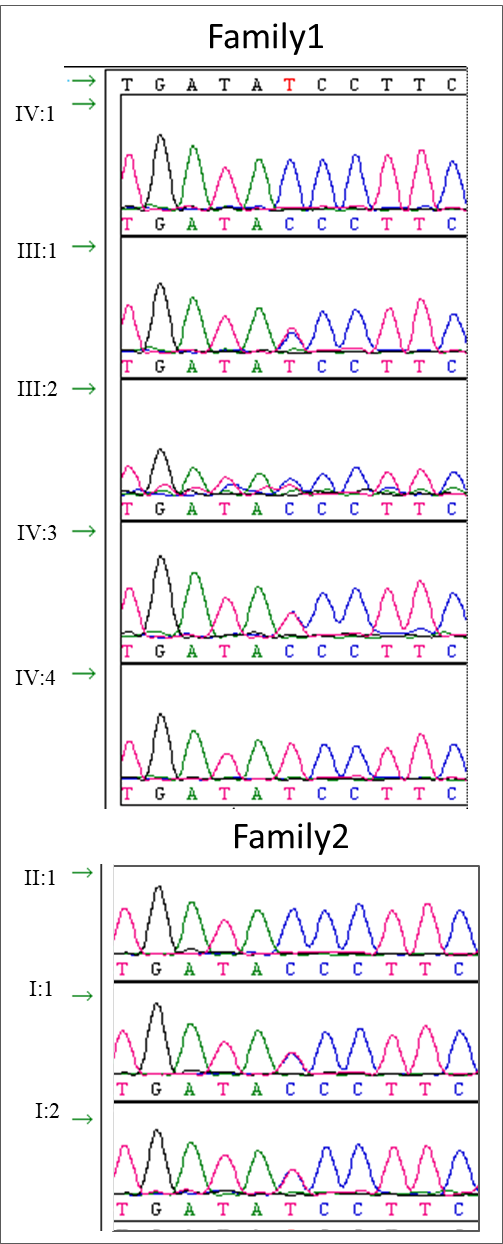

Supplement: Supplementary file 1 — Supplementary figure 1. [file 41439_2023_234_MOESM1_ESM.png]
